# Supplementary material for: Ultrasound-activated piezo-hot carriers trigger tandem catalysis coordinating cuproptosis-like bacterial death against implant infections
Source: Nat Commun. 2024 Feb 22;15:1643. doi: 10.1038/s41467-024-45619-y (PMC10884398; doi:10.1038/s41467-024-45619-y)
Supplement: Supplementary file 1 — Supplementary Information [file 41467_2024_45619_MOESM1_ESM.pdf]

Supporting information

Ultrasound-Activated Piezo-Hot Carriers Trigger Tandem Catalysis Coordinating Cuproptosis-Like Bacterial Death Against Implant Infections

Yanli Huang[1], Xufeng Wan[2], Qiang Su[3], Chunlin Zhao[4], Jian Cao[2], Yan Yue[2], Shuoyuan Li[2], Xiaoting Chen[5], Jie Yin[6], Yi Deng[7], Xianzeng Zhang[1,*], Tianmin Wu[1,*], Zongke Zhou[2,*], Duan Wang[2,*]

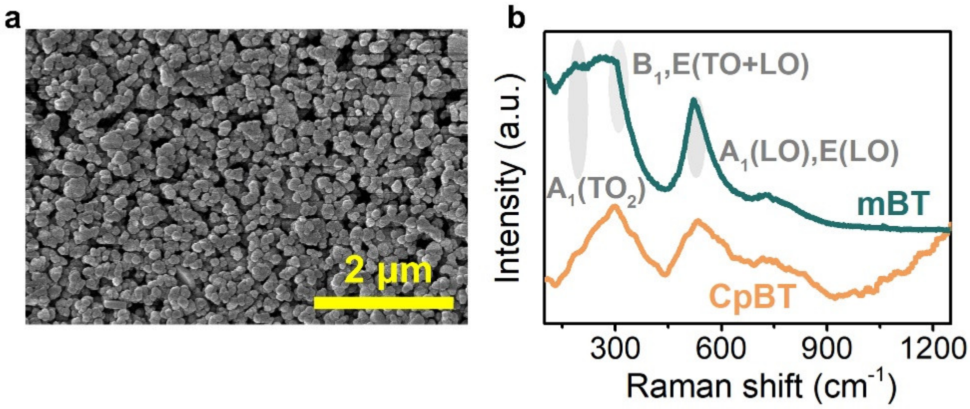

Fig. S1 a The SEM images of CpBT. Similar SEM images were obtained for more than three times experiments. **b** Raman spectra of mBT and CpBT.

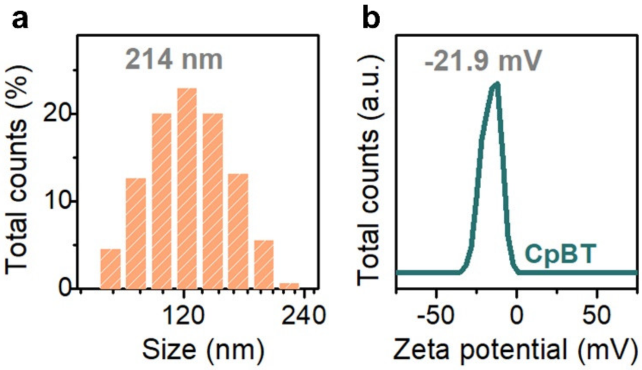

Fig. S2 a Size distribution and **b** Zeta potential of CpBT by DLS.

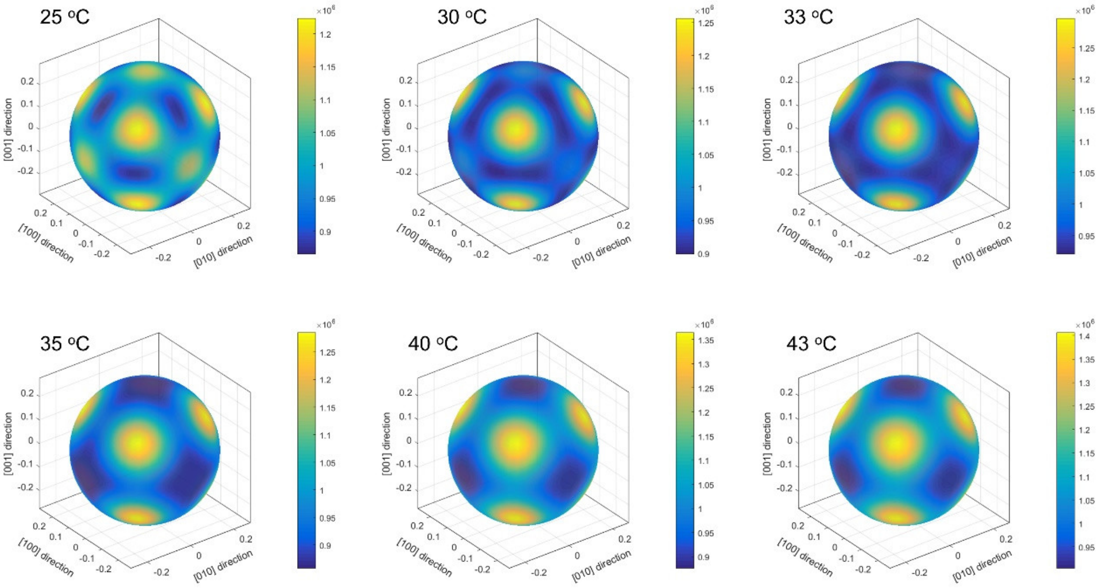

Fig. S3 Free-energy profiles for mBT at different temperatures.

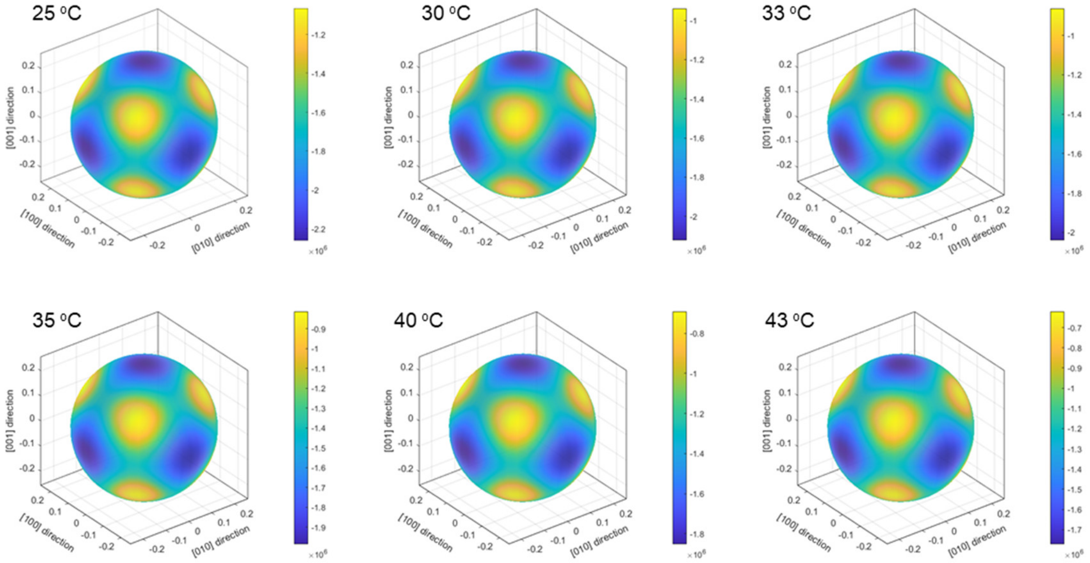

Fig. S4 The free-energy profiles for pure BT at different temperatures.

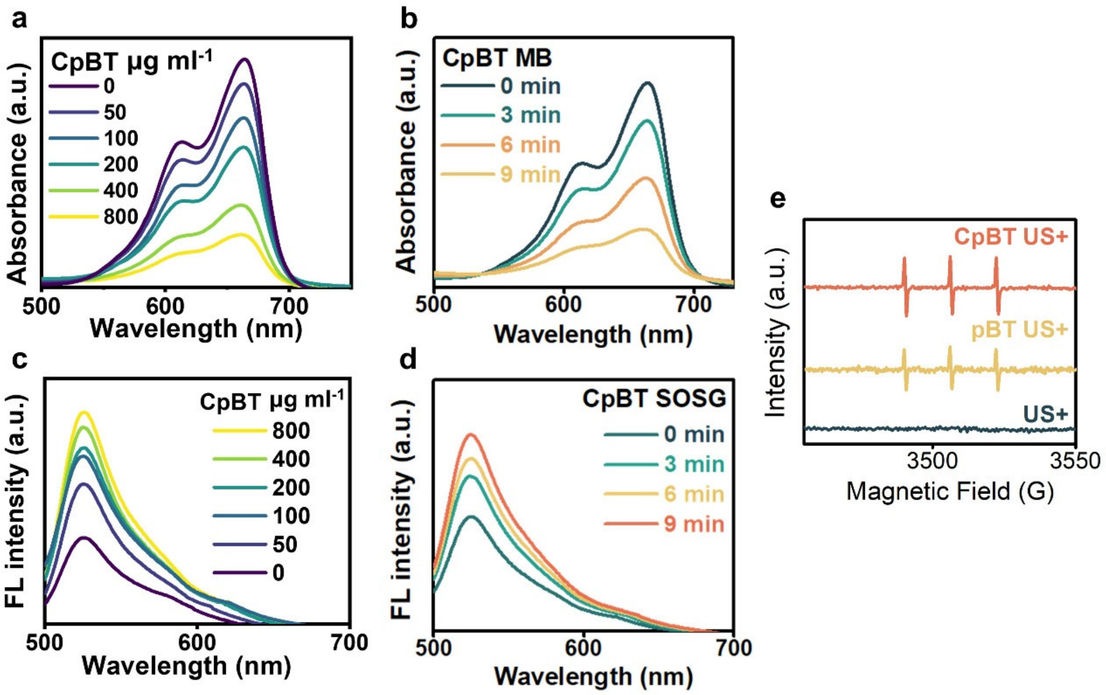

Fig. S5 MB degradation by CpBT with **a** different concentration and **b** different time under US irradiation. Fluorescence intensity of SOSG treated with CpBT under US irradiation with **c** different concentration and **d** different time. **e** $^1O_2$ generation by ESR.

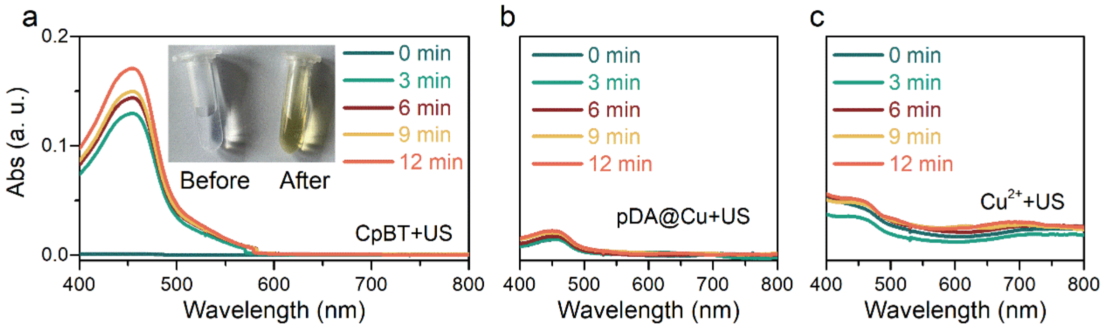

Fig. S6 UV–Vis absorbance spectra of neocuproine treated with **a** CpBT+US stimulation, **b** pDA@Cu+US (pDA nanoparticles with Cu^{2+} chelating on the surface), and **c** Cu^{2+} ions+US (Cu^{2+} ions were from $CuSO_4$).

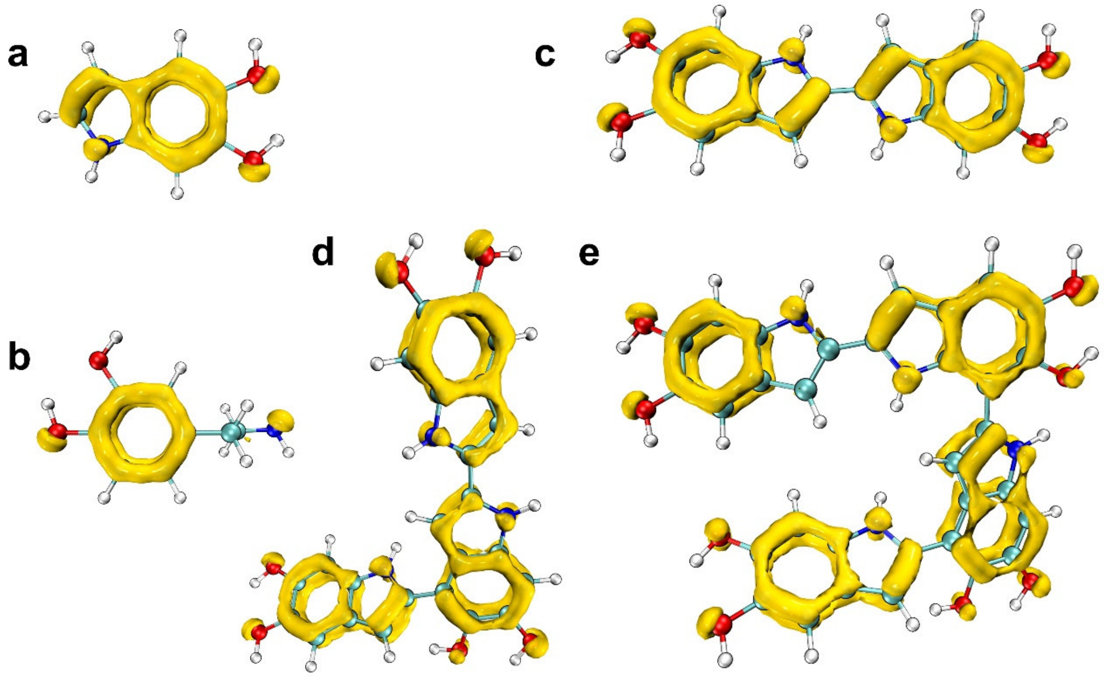

Fig. S7 LOL-π isosurface of **a** DHI, **b** DA, **c** DHI dimer, **d** DHI trimer, and **e** DHI tetramer. Isosurface value was set to be 0.5 e/Å^3.

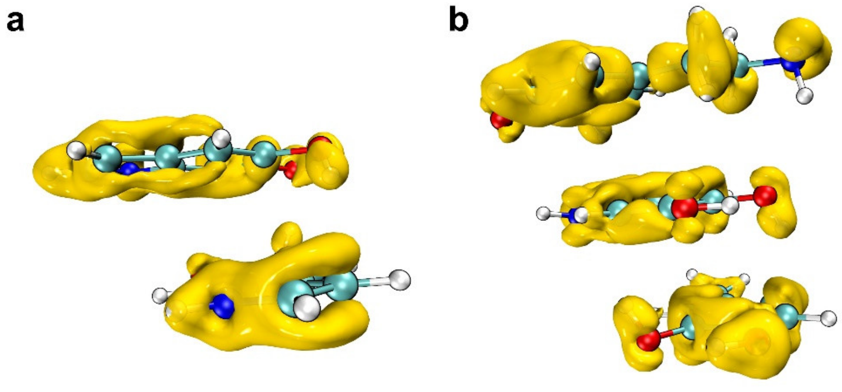

Fig. S8 Isosurface map of LOL-π for **a** two-layer stacked DHI and **b** three-layer stacked DHI and DA. Isosurface value is set to be 0.5 e/Å^3.

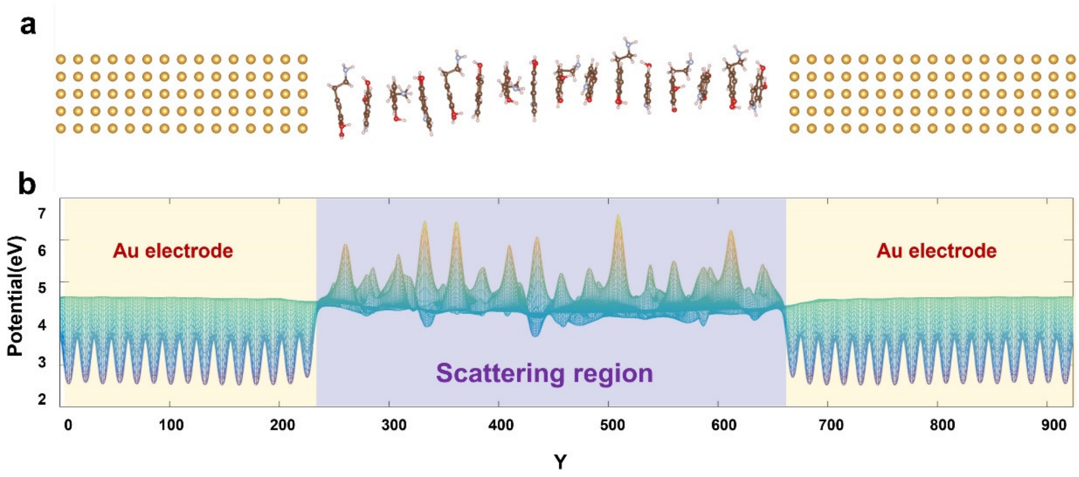

Fig. S9 a Side view of the atomic structure of the 16-layer stacked DHI and DA-based quantum transport architecture device. **b** The potential energy distribution along XY-plane.

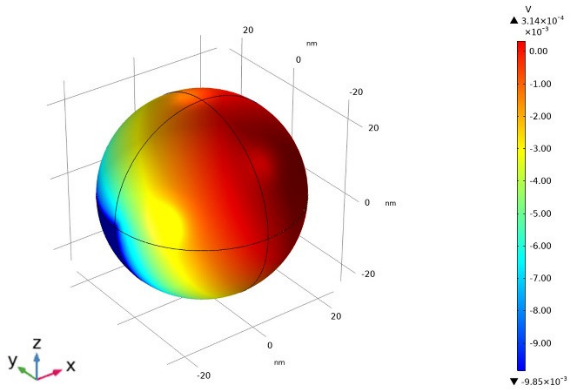

Fig. S10 Finite element method simulation for piezo-potential distribution on the surface of pure BT with the cavitation pressure of 10^8 Pa.

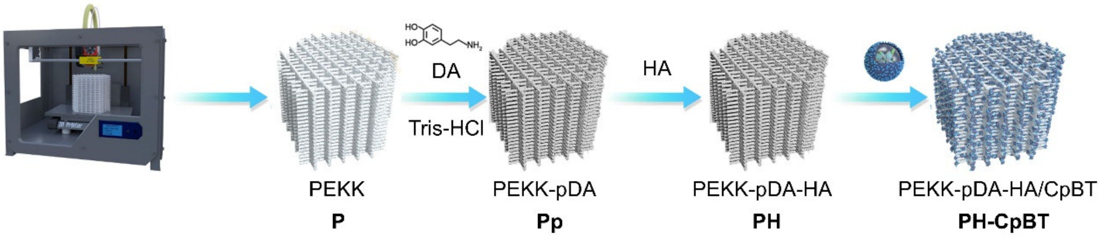

Fig. S11 The fabrication process of PH-CpBT bone scaffold.

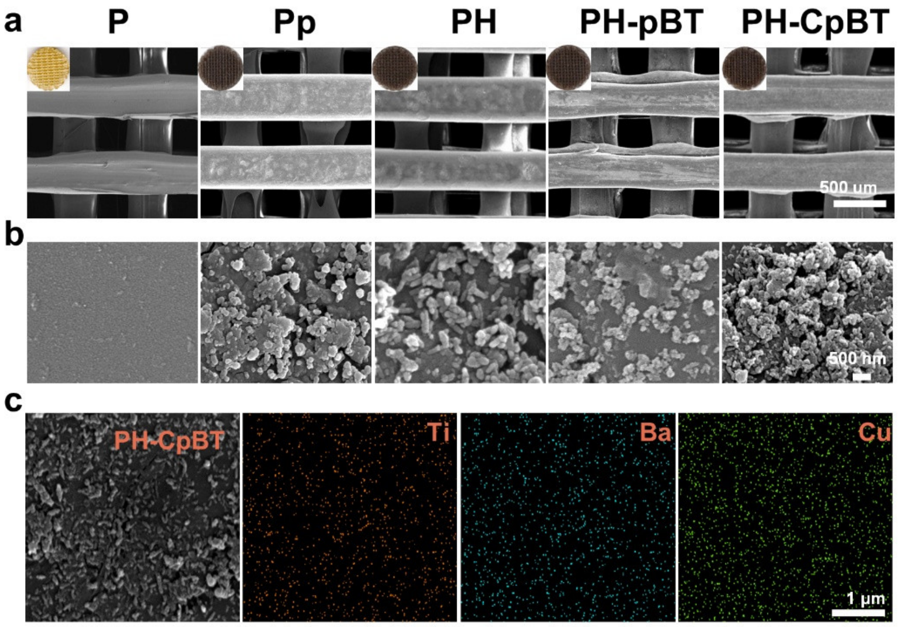

Fig. S12 a SEM images and **b** the corresponding enlarged view for different scaffolds. **c** The element mapping of Ti, Ba, and Cu for PH-CpBT. Similar SEM images were obtained for more than three times experiments.

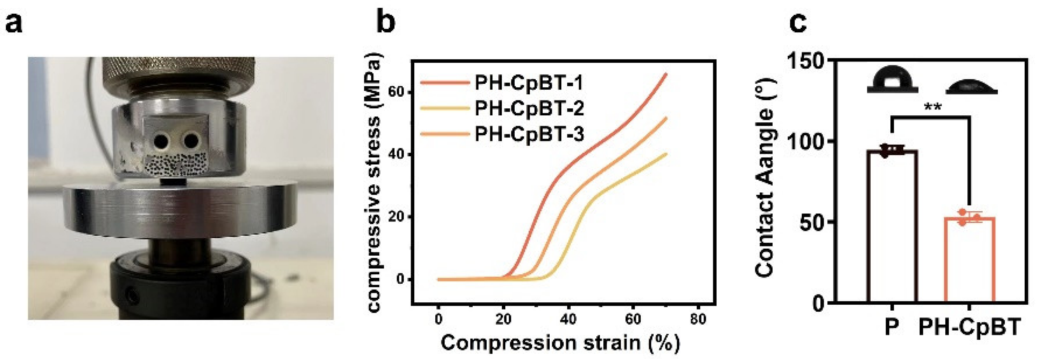

Fig. S13 a The image of PH-CpBT during measurement. **b** Young's modulus, and **c** contact angle for PH-CpBT, n = 3 independent samples; unpaired t test; data were presented as mean values ± SD; error bars = SD; $^{**}p < 0.01$.

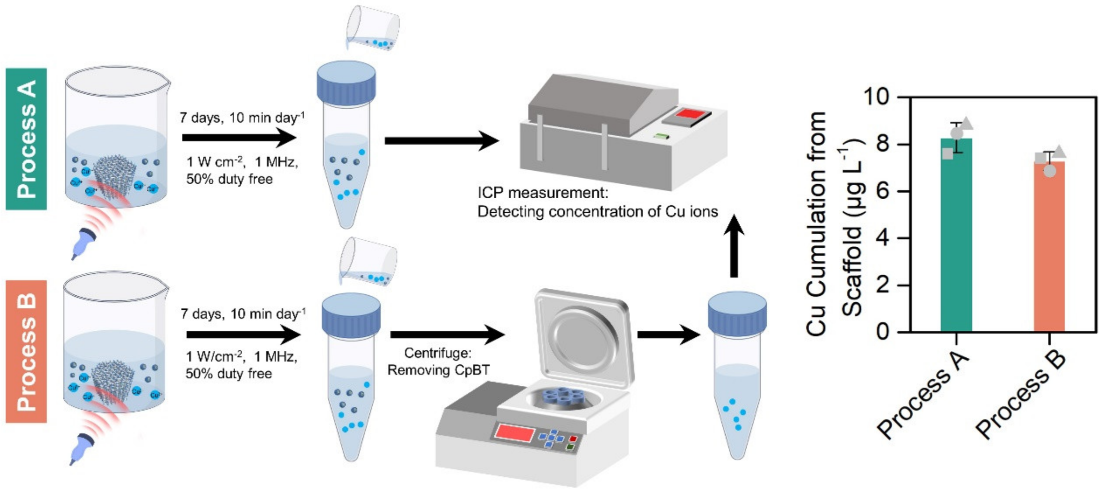

Fig. S14 Cu ions released from PH-CpBT scaffold with different treatments by ICP, n = 3 independent samples, data were presented as mean values ± SD, error bars = SD.

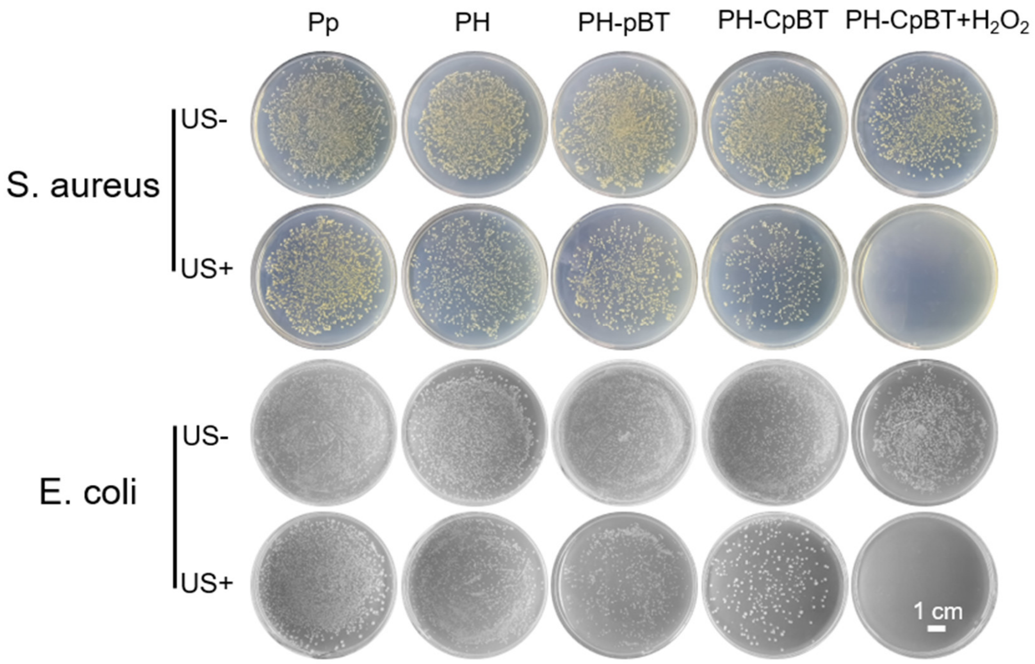

Fig. S15 Typical images of *S. aureus* and *E. coli* colonies treated by various groups. A representative image of three biological replicates from each group was shown.

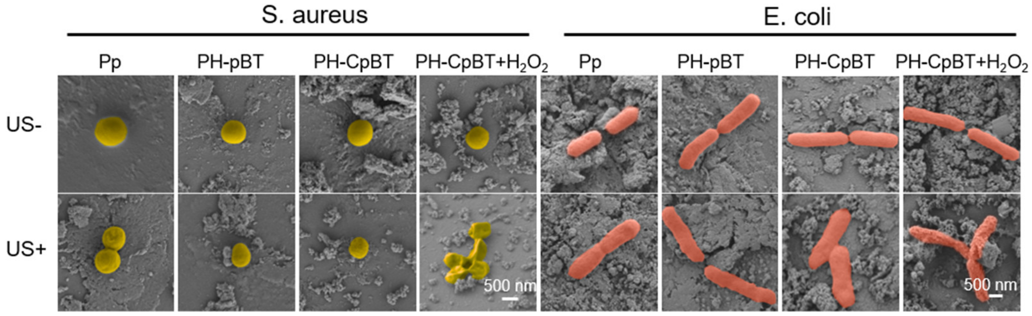

Fig. S16 SEM images of *S. aureus* and *E. coli* on different scaffolds with or without US irradiation. A representative image of three biological replicates from each group was shown.

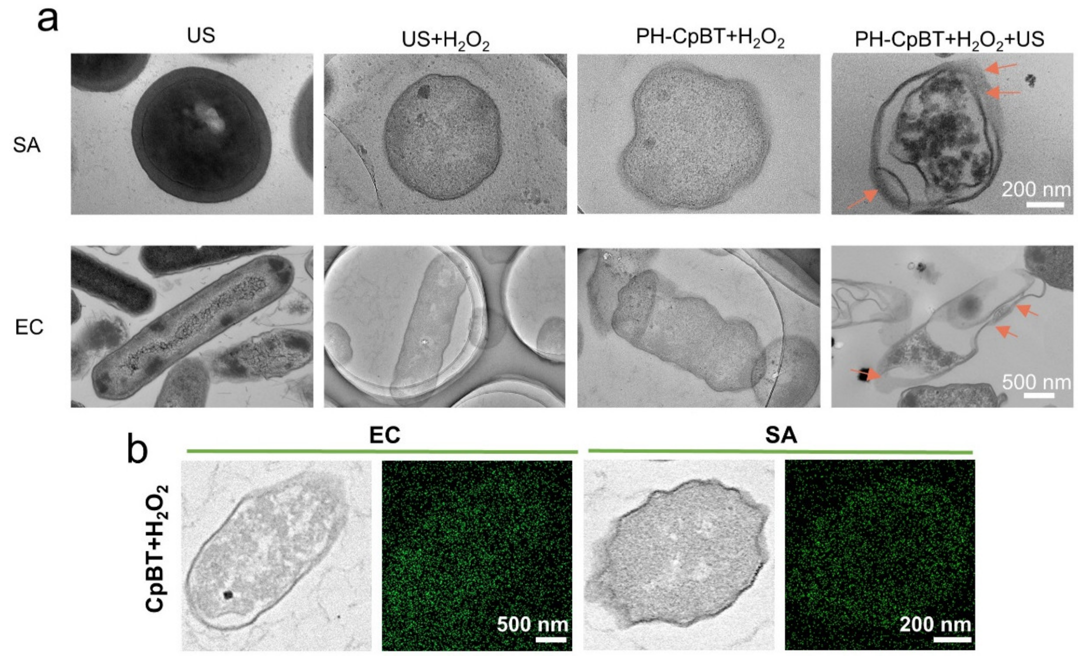

Fig. S17 **a** The microstructure of *S. aureus* and *E. coli* treated by different groups observed by Bio-TEM. **b** The element mapping of Cu in bacteria treated with CpBT+H_2O_2. A representative image of three biological replicates from each group was shown.

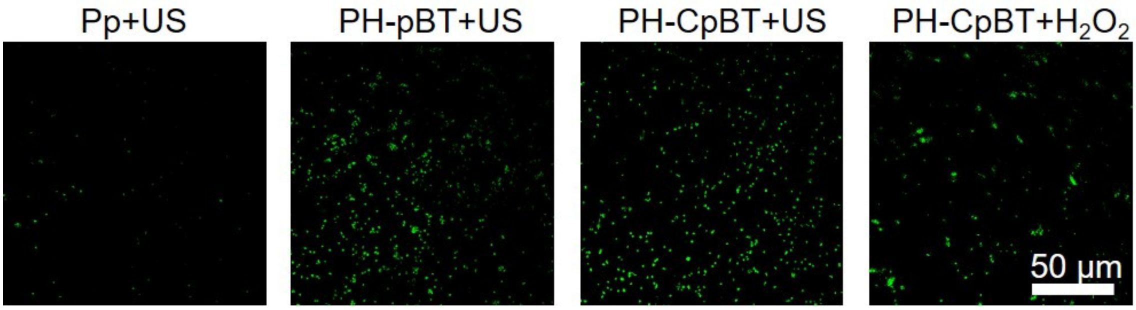

Fig. S18 The fluorescence image of intracellular ROS in *S. aureus* with DCFH probe. A representative image of three biological replicates from each group was shown.

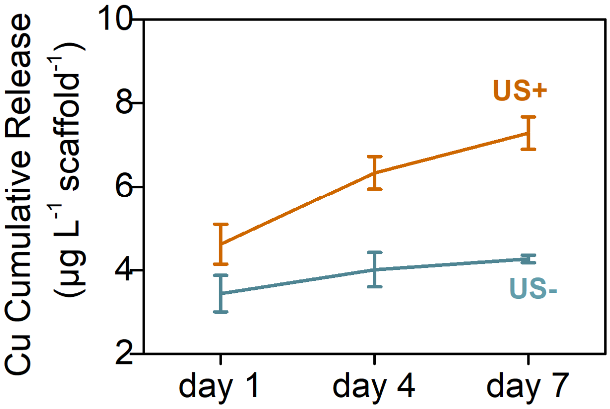

Fig. S19 Cu ions released from PH-CpBT scaffold in the 0.9 % NaCl solution at different immersion time with or without US stimulation (10 min day^{-1}, 1 W cm^{-2}, 1 MHz). Data were presented as mean values ± SD; error bars = SD.

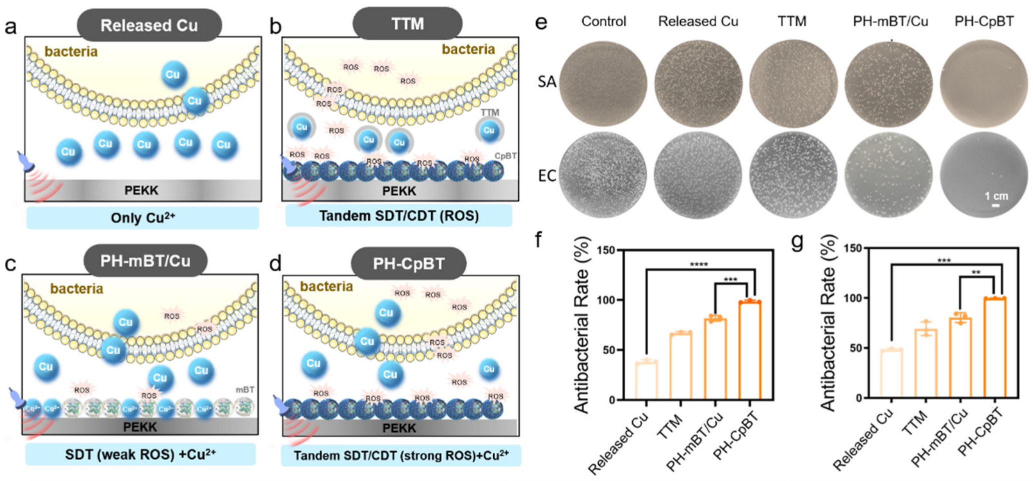

Fig. S20 a-d Schematic diagram for different antibacterial experiment. **e** Typical images of *S. aureus* and *E. coli* colonies treated by various groups. A representative image of three biological replicates from each group was shown. The corresponding strain counts of spread plate against **f** *S. aureus* and **g** *E. coli* after different treatments. **f, g** $n = 3$ independent samples; ANOVA followed by Tukey's multiple comparisons; data are presented as mean values ± SD; error bars = SD. Significant differences between groups were indicated as ****$p < 0.001$, ***$p < 0.001$, **$p < 0.01$, and *$p < 0.05$.

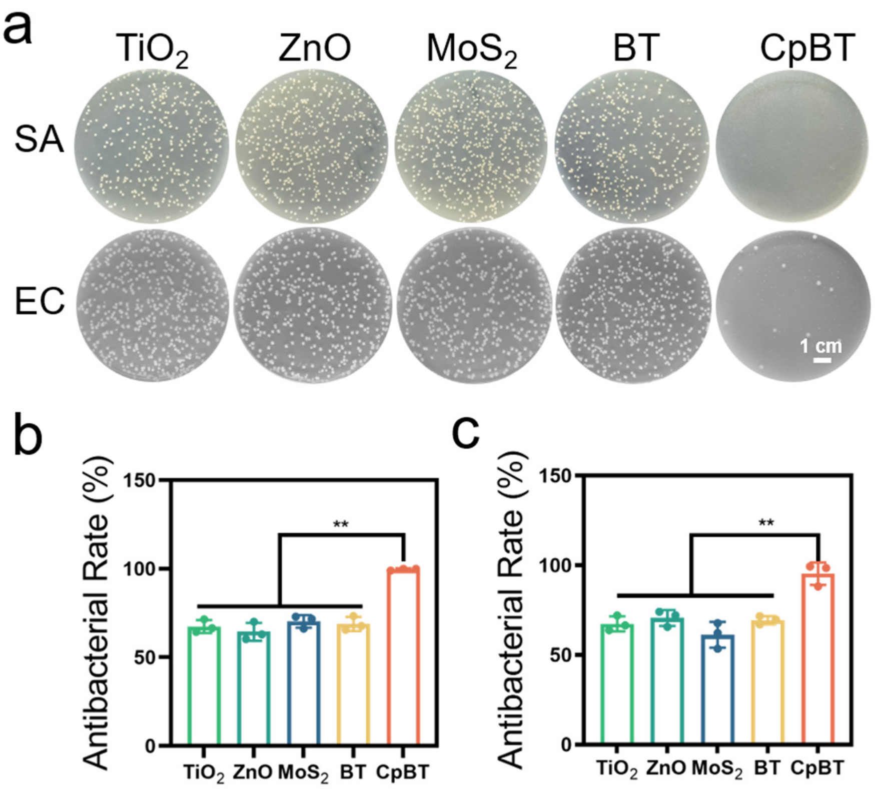

Fig. S21 a Plate cultures of *S. aureus* and *E. coli* treat with different materials and US stimulation. A representative image of three biological replicates from each group was shown. The related antibacterial rate against **b** *S. aureus* and **c** *E. coli*. $n = 3$ independent samples; ANOVA followed by Tukey's multiple comparisons; data were presented as mean values ± SD; error bars = SD. Significant differences between groups were indicated as $^{**}p < 0.01$.

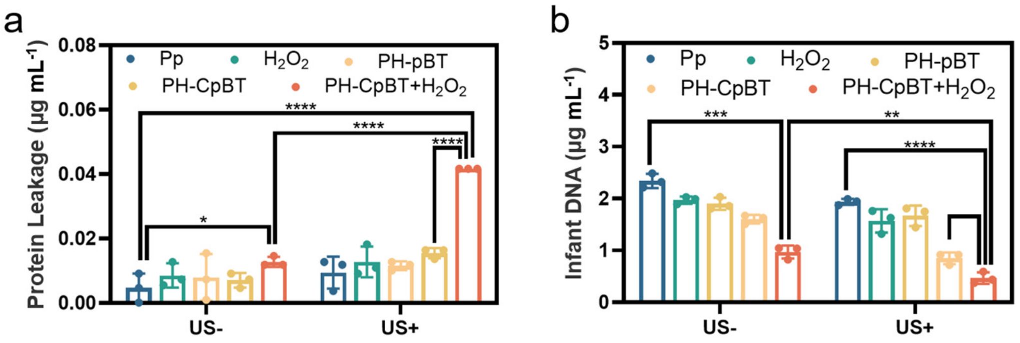

Fig. S22 Quantitative analysis of **a** protein leakage and **b** the intact bacterial DNA of *S. aurues* on different scaffolds after different treatments, n = 3 independent samples; ANOVA followed by Tukey's multiple comparisons; data are presented as mean values ± SD; error bars = SD. Significant differences between groups were indicated as ****p < 0.001, ***p < 0.001, **p < 0.01, and *p < 0.05.

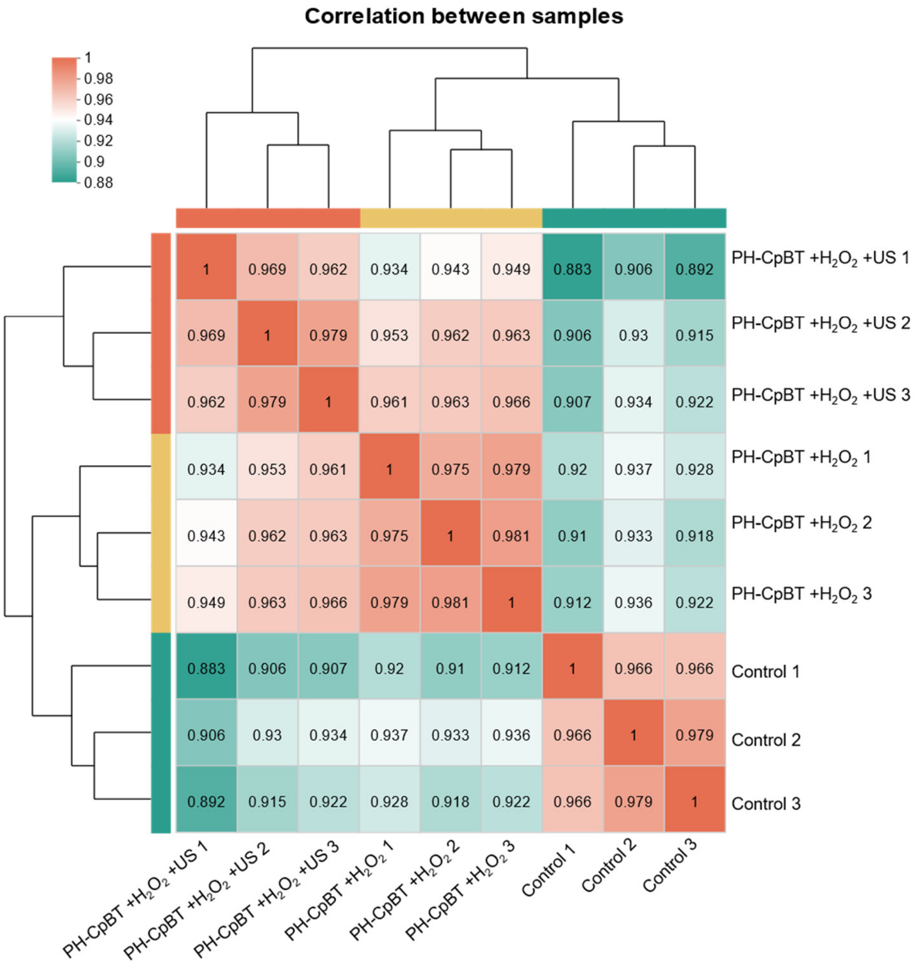

Fig. S23 Heat map to show the correlation between samples.

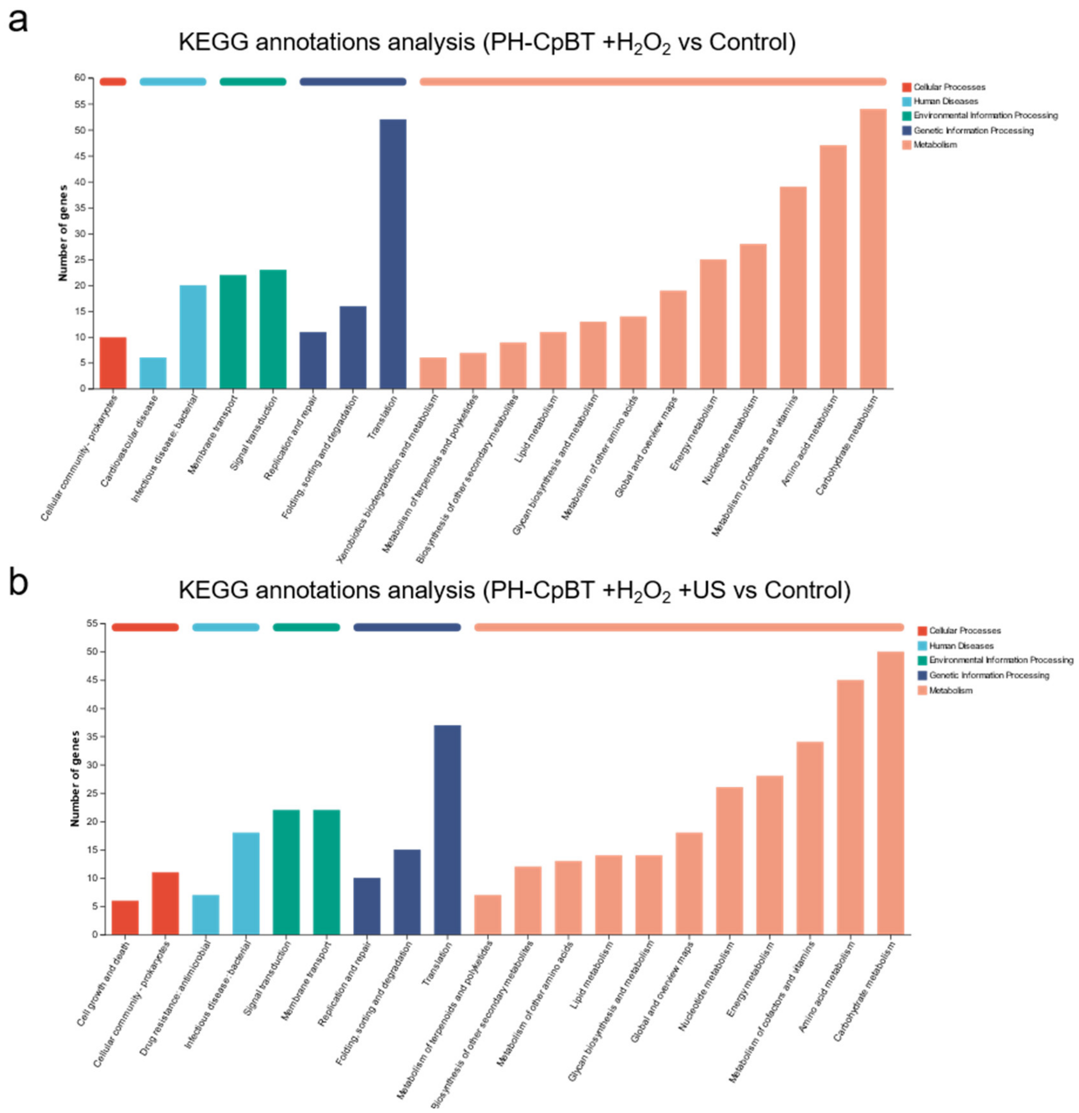

Fig. S24 The KEGG annotations of DEGs in response to **a** PH-CpBT +H_2O_2 and **b** PH-CpBT +H_2O_2 +US.

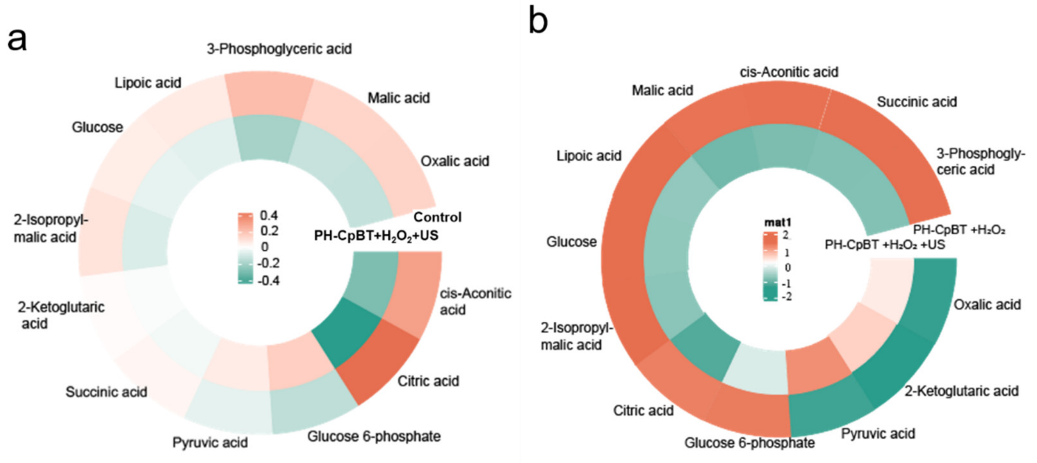

Fig. S25 Differential heat map of TCA cycle metabolites. **a** PH-CpBT+H_2O_2+US *vs.* Control. **b** PH-CpBT+H_2O_2+US *vs.* PH-CpBT+H_2O_2. **a, b** $n = 6$ independent samples; unpaired t test; $p < 0.05$.

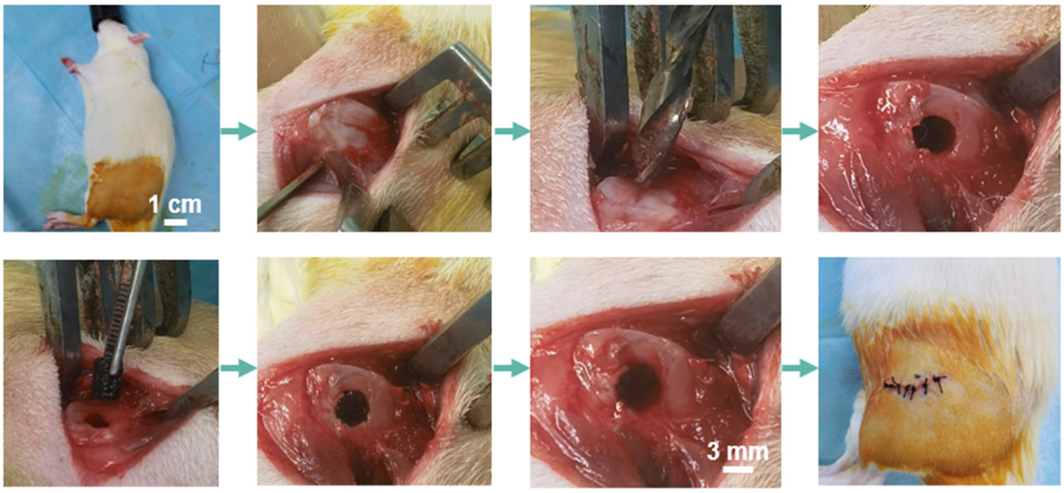

Fig. S26 Rat femoral condyle defect modeling and PEKK scaffolds implantation.

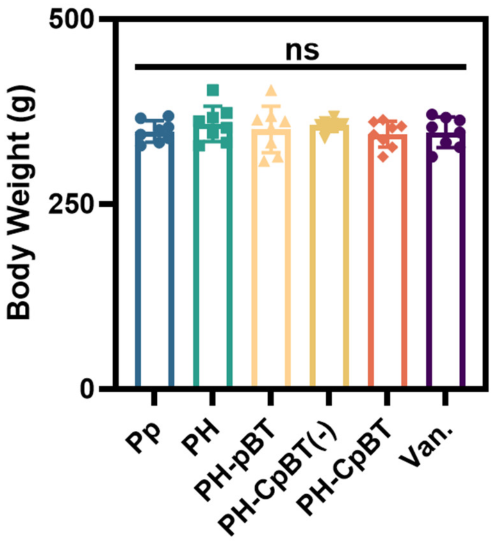

Fig. S27 The initial body weight of rats for different groups, $n = 8$ independent samples; ANOVA followed by Tukey's multiple comparisons; data were presented as mean values ± SD; error bars = SD. Significant differences between groups were indicated as ****$p < 0.001$, ***$p < 0.001$, **$p < 0.01$, and *$p < 0.05$.

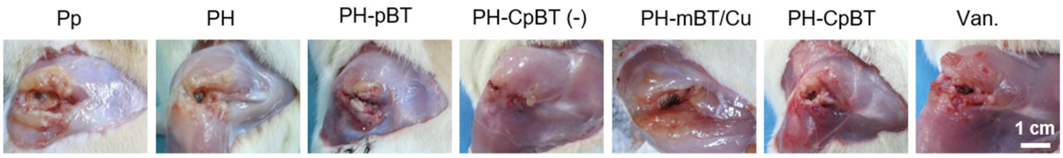

Fig. S28 The macroscopic observation of knee-joint post-implantation showing the infections. A representative image of eight biological replicates from each group was shown.

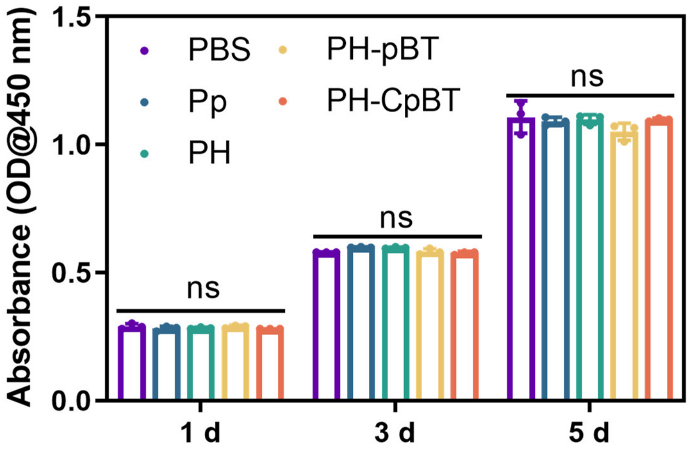

Fig. S29 Cytotoxicity of different scaffolds, $n = 3$ independent samples; ANOVA followed by Tukey's multiple comparisons; data were presented as mean values ± SD; error bars = SD. Significant differences between groups were indicated as ****$p < 0.001$, ***$p < 0.001$, **$p < 0.01$, and *$p < 0.05$.

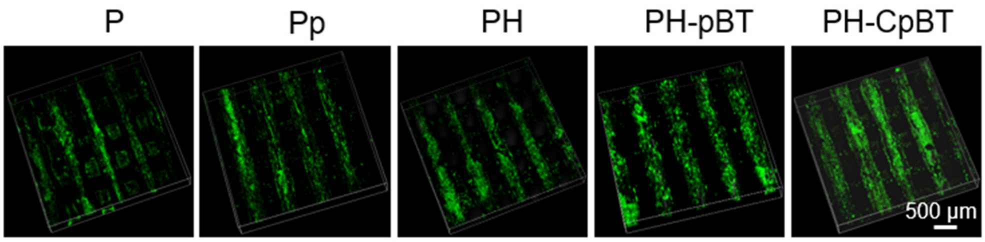

Fig. S30 Fluorescence images of Live/Dead staining of MC3T3-E1 cells on scaffolds. A representative image of three biological replicates from each group was shown.

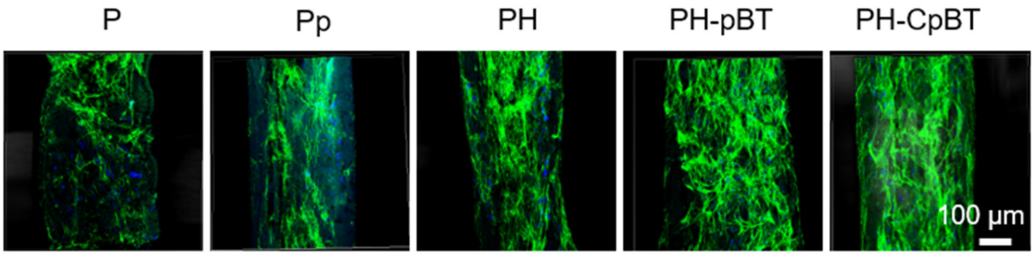

Fig. S31 Fluorescence images of MC3T3-E1 cultured on different scaffolds, the cytoskeletons stained with FITC (green) and the nuclei stained with DAPI (blue). A representative image of three biological replicates from each group was shown.

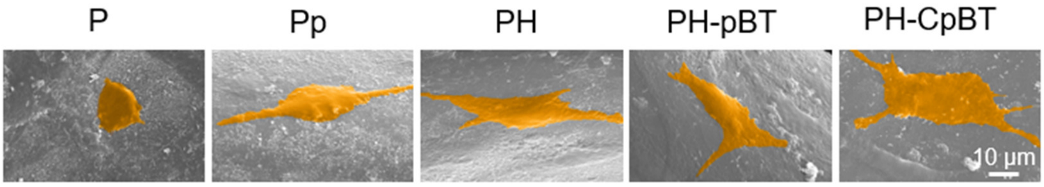

Fig. S32 Morphologies of cells cultured on different scaffolds captured by SEM. A representative image of three biological replicates from each group was shown.

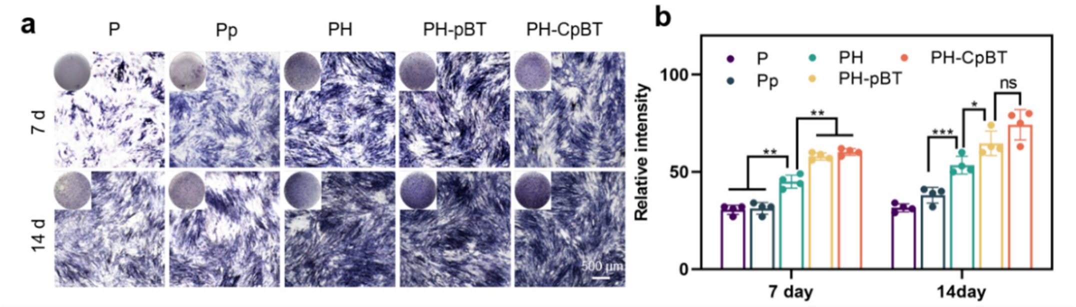

Fig. S33 **a** Photographs and **b** semi-quantification of ALP staining on the scaffolds, $n = 4$ independent samples; ANOVA followed by Tukey's multiple comparisons; data were presented as mean values ± SD; error bars = SD. Significant differences between groups were indicated as ****$p < 0.001$, ***$p < 0.001$, **$p < 0.01$, and *$p < 0.05$.

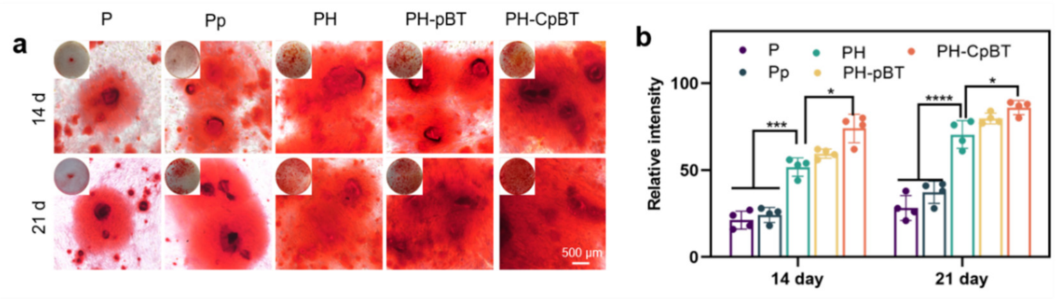

Fig. S34 **a** Photographs and **b** semi-quantification of ARS staining on the scaffolds, $n = 4$ independent samples; ANOVA followed by Tukey's multiple comparisons; data were presented as mean values ± SD; error bars = SD. Significant differences between groups were indicated as ****$p < 0.001$, ***$p < 0.001$, **$p < 0.01$, and *$p < 0.05$.

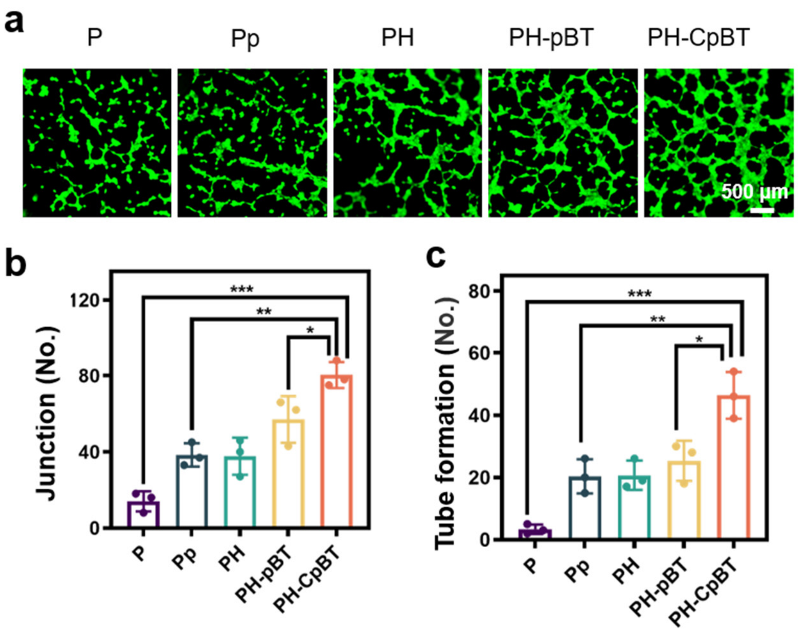

Fig. S35 a The tubulogenesis of HUVEC cells stained with calcein. A representative image of three biological replicates from each group was shown. Semi-quantification of the number of **b** junctions **c** and tube formations, $n = 3$ independent samples; ANOVA followed by Tukey's multiple comparisons; data were presented as mean values ± SD; error bars = SD. Significant differences between groups were indicated as *****p* < 0.001, ****p* < 0.001, ***p* < 0.01, and **p* < 0.05.

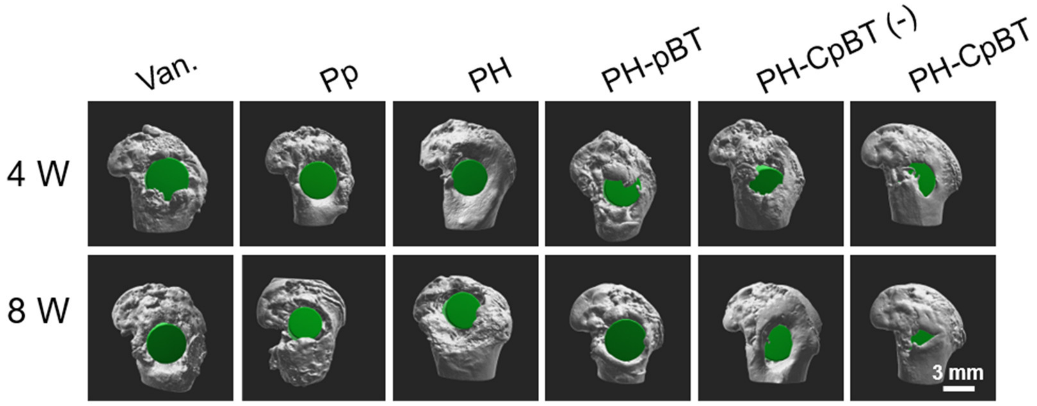

Fig. S36 The 3D reconstruction of knee-joint post-implantation by Imaris. A representative image of four (4W) and six (8W) biological replicates from each group was shown.

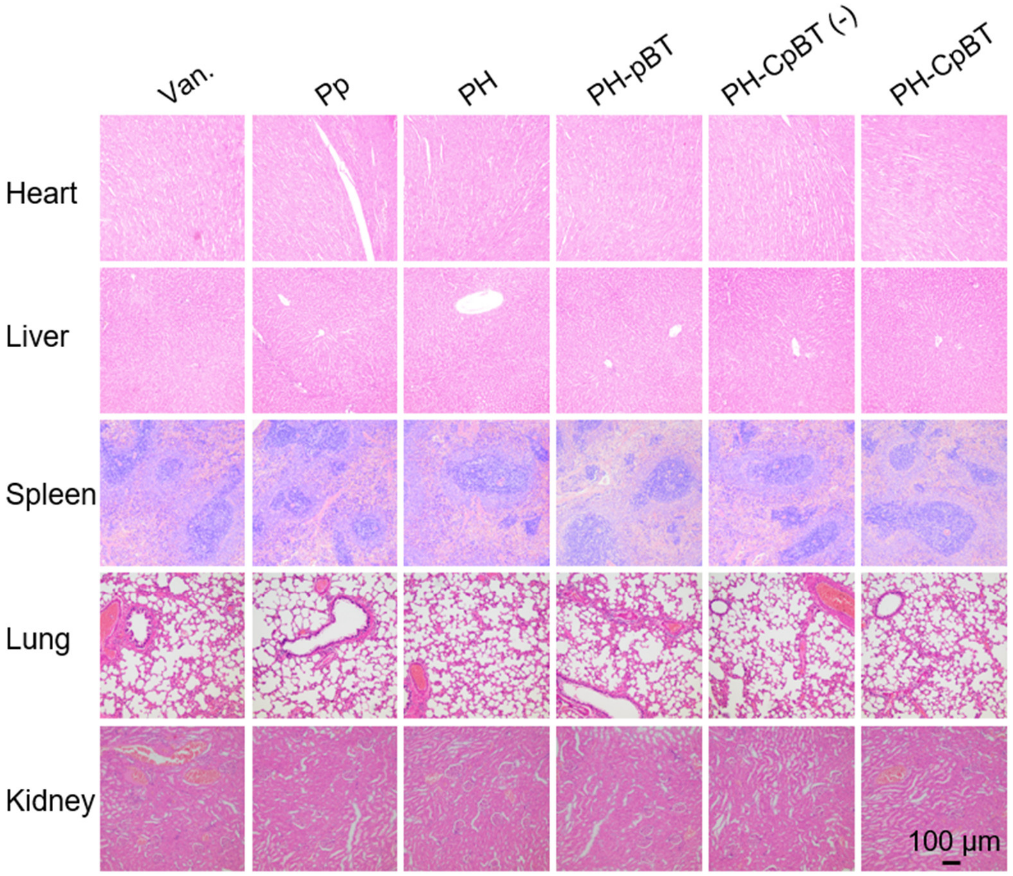

Fig. S37 The toxicity of main organs by H&E staining. A representative image of three biological replicates from each group was shown.

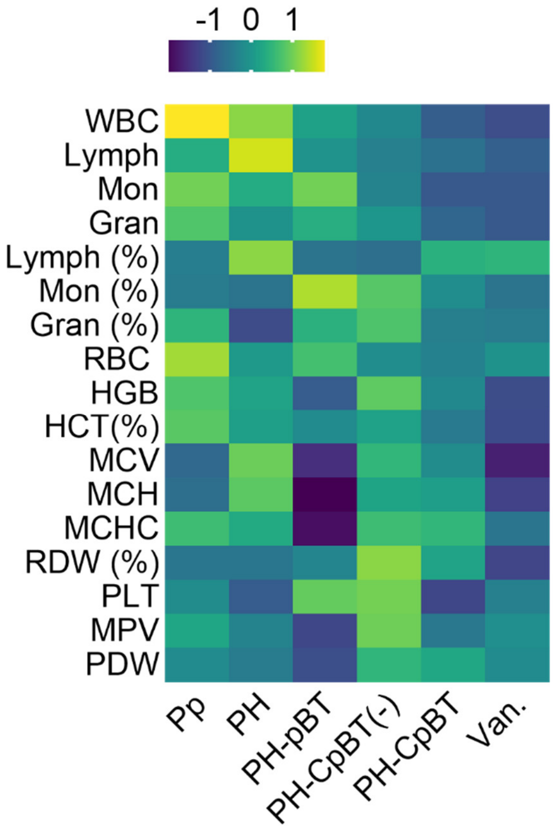

Fig. S38 Heat map of blood routine.

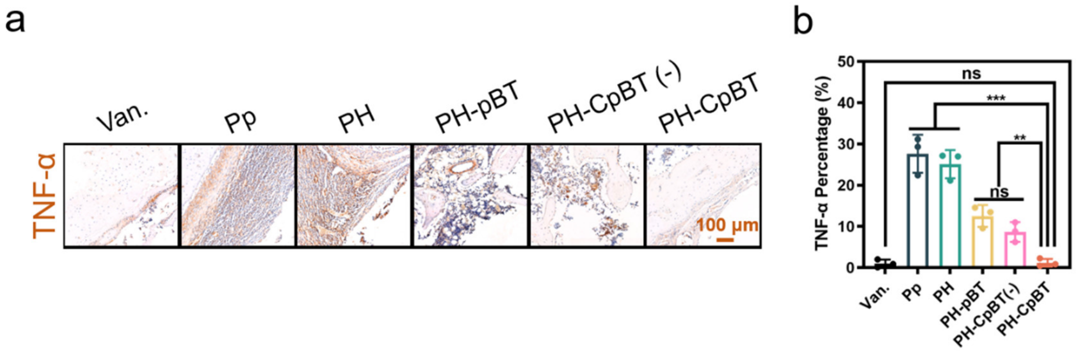

Fig. S39 Immunohistochemistry image of **a** TNF-α and **b** its quantitation. A representative image of three biological replicates from each group was shown. **b** $n = 3$ independent samples; ANOVA followed by Tukey's multiple comparisons; data were presented as mean values ± SD; error bars = SD. Significant differences between groups were indicated as ****$p < 0.001$, ***$p < 0.001$, **$p < 0.01$, and *$p < 0.05$.
