# Supplementary material for: Ultrasound-activated piezo-hot carriers trigger tandem catalysis coordinating cuproptosis-like bacterial death against implant infections
Source: Nat Commun. 2024 Feb 22;15:1643. doi: 10.1038/s41467-024-45619-y (PMC10884398; doi:10.1038/s41467-024-45619-y)
Supplement: Supplementary file 3 — Description of Additional Supplementary Files [file 41467_2024_45619_MOESM3_ESM.pdf]

Description of Additional Supplementary Files

1. **Supplementary Movie 1** Description: 3D reconstruction by micro-CT
