# Supplementary material for: Ultrasound-activated piezo-hot carriers trigger tandem catalysis coordinating cuproptosis-like bacterial death against implant infections
Source: Nat Commun. 2024 Feb 22;15:1643. doi: 10.1038/s41467-024-45619-y (PMC10884398; doi:10.1038/s41467-024-45619-y)
Supplement: Supplementary file 5 — Reporting Summary [file 41467_2024_45619_MOESM5_ESM.pdf]

nature portfolio

Corresponding author(s): Xianzeng Zhang, Tianmin Wu, Zongke Zhou, and Duan Wang

Last updated by author(s): Jan 15, 2024

Reporting Summary

Nature Portfolio wishes to improve the reproducibility of the work that we publish. This form provides structure for consistency and transparency in reporting. For further information on Nature Portfolio policies, see our Editorial Policies and the Editorial Policy Checklist.

Statistics

For all statistical analyses, confirm that the following items are present in the figure legend, table legend, main text, or Methods section.

n/a	Confirmed	
☐	☒	The exact sample size (*n*) for each experimental group/condition, given as a discrete number and unit of measurement
☐	☒	A statement on whether measurements were taken from distinct samples or whether the same sample was measured repeatedly
☐	☒	The statistical test(s) used AND whether they are one- or two-sided<br>*Only common tests should be described solely by name; describe more complex techniques in the Methods section.*
☒	☐	A description of all covariates tested
☒	☐	A description of any assumptions or corrections, such as tests of normality and adjustment for multiple comparisons
☐	☒	A full description of the statistical parameters including central tendency (e.g. means) or other basic estimates (e.g. regression coefficient) AND variation (e.g. standard deviation) or associated estimates of uncertainty (e.g. confidence intervals)
☐	☒	For null hypothesis testing, the test statistic (e.g. *F*, *t*, *r*) with confidence intervals, effect sizes, degrees of freedom and *P* value noted<br>*Give P values as exact values whenever suitable.*
☒	☐	For Bayesian analysis, information on the choice of priors and Markov chain Monte Carlo settings
☒	☐	For hierarchical and complex designs, identification of the appropriate level for tests and full reporting of outcomes
☒	☐	Estimates of effect sizes (e.g. Cohen's *d*, Pearson's *r*), indicating how they were calculated

Our web collection on statistics for biologists contains articles on many of the points above.

Software and code

Policy information about availability of computer code

Data collection	The data were analysed by using Origin 9.0, Microsoft Excel 2021, and GraphPad Prism 8.0.
Data analysis	The data were analysed by Materialise 3-Matic, Nanodcal, DESeq2 (Version 1.24.0), Origin 9.0, Microsoft Excel 2021, GraphPad Prism 8.0, Gaussian16 program, Image J, Skyscan NRecon, Sciex OS, and Imaris software. The details are provided in Methods.

For manuscripts utilizing custom algorithms or software that are central to the research but not yet described in published literature, software must be made available to editors and reviewers. We strongly encourage code deposition in a community repository (e.g. GitHub). See the Nature Portfolio guidelines for submitting code & software for further information.

Data

Policy information about availability of data

All manuscripts must include a data availability statement. This statement should provide the following information, where applicable:

- Accession codes, unique identifiers, or web links for publicly available datasets
- A description of any restrictions on data availability
- For clinical datasets or third party data, please ensure that the statement adheres to our policy

All data supporting the findings of this study are available within the article and the Supplementary Information. Source Data are provided with this paper. The RNA-seq data generated in this study are available on the National Center for Biotechnology Information (NCBI) database under the BioProject PRJNA1064643 (https://www.ncbi.nlm.nih.gov/bioproject/PRJNA1064643).

Research involving human participants, their data, or biological material

Policy information about studies with human participants or human data. See also policy information about sex, gender (identity/presentation), and sexual orientation and race, ethnicity and racism.

Reporting on sex and gender	The sex or gender in this work is not considered.
Reporting on race, ethnicity, or other socially relevant groupings	*Please specify the socially constructed or socially relevant categorization variable(s) used in your manuscript and explain why they were used. Please note that such variables should not be used as proxies for other socially constructed/relevant variables (for example, race or ethnicity should not be used as a proxy for socioeconomic status).*<br>*Provide clear definitions of the relevant terms used, how they were provided (by the participants/respondents, the researchers, or third parties), and the method(s) used to classify people into the different categories (e.g. self-report, census or administrative data, social media data, etc.)*<br>*Please provide details about how you controlled for confounding variables in your analyses.*
Population characteristics	*Describe the covariate-relevant population characteristics of the human research participants (e.g. age, genotypic information, past and current diagnosis and treatment categories). If you filled out the behavioural & social sciences study design questions and have nothing to add here, write "See above."*
Recruitment	*Describe how participants were recruited. Outline any potential self-selection bias or other biases that may be present and how these are likely to impact results.*
Ethics oversight	*Identify the organization(s) that approved the study protocol.*

Note that full information on the approval of the study protocol must also be provided in the manuscript.

Field-specific reporting

Please select the one below that is the best fit for your research. If you are not sure, read the appropriate sections before making your selection.

☒ Life sciences ☐ Behavioural & social sciences ☐ Ecological, evolutionary & environmental sciences

For a reference copy of the document with all sections, see nature.com/documents/nr-reporting-summary-flat.pdf

Life sciences study design

All studies must disclose on these points even when the disclosure is negative.

Sample size	Each finding was confirmed with minimum necessary number such as 3-6 replicates for each experiments. The sample was randomly choose to be determined. These sample sizes are sufficient which have reached statistical significance.
Data exclusions	No data were excluded from the analyses.
Replication	All experiments were performed with independent replicates as described in the figure legends.
Randomization	To ensure the scientific difference between the experimental groups and control groups, the samples that have the similar properties (e.g., the age and weight of rats) to the control, were randomly allocated into experimental groups.
Blinding	No blinding was applied. The data analyses are based on objectively measurable data, so the investigators were not blinded to allocation during experiments and outcome assessment.

Reporting for specific materials, systems and methods

We require information from authors about some types of materials, experimental systems and methods used in many studies. Here, indicate whether each material, system or method listed is relevant to your study. If you are not sure if a list item applies to your research, read the appropriate section before selecting a response.

Materials & experimental systems

n/a	Involved in the study
☐	☒ Antibodies
☐	☒ Eukaryotic cell lines
☒	☐ Palaeontology and archaeology
☐	☒ Animals and other organisms
☒	☐ Clinical data
☒	☐ Dual use research of concern
☒	☐ Plants

Methods

n/a	Involved in the study
☒	☐ ChIP-seq
☒	☐ Flow cytometry
☒	☐ MRI-based neuroimaging

Antibodies

Antibodies used	DAPI (abcam, ab285390)<br>BMP-2 (abcam, ab214821)<br>RUNX2 (abcam, ab236639)<br>CD31 (abcam, ab182981)<br>TNF-α (abcam, ab1793)
Validation	All the commercial antibodies have been validated by manufactures and the statements can be found on the manufactures' websites. Antibodies were all validated by positive control experiments.

Eukaryotic cell lines

Policy information about cell lines and Sex and Gender in Research

Cell line source(s)	Mouse osteoblastic MC3T3-E1 cells (GNM15) were purchased from the Cell Bank of the Chinese Academy of Sciences (Shanghai, China). The HUVECs (CP-H082) were purchased from Procell Life Science & Technology Co., LTD. (Wuhan, China).
Authentication	No further authentication was done after the cells were obtained from the vendors.
Mycoplasma contamination	All cell lines are tested negative for Mycoplasma contamination.
Commonly misidentified lines (See ICLAC register)	None misidentified lines were used.

Animals and other research organisms

Policy information about studies involving animals; ARRIVE guidelines recommended for reporting animal research, and Sex and Gender in Research

Laboratory animals	Male Sprague Dawley rats (250–300 g, about two-months old) were purchased from the Beijing Huafukang Bioscience Cojnc.
Wild animals	The study did not involve wild animals.
Reporting on sex	The sex difference was not important for in vivo experiments including biosafety study, biodistribution study and antibacterial efficacy study. Therefore, in this study we performed the all animal experiments using male rats.
Field-collected samples	There are no samples collected from the field in this study.
Ethics oversight	Animal experimentation in this study received ethical approval from the Laboratory Animal Ethics Committee of West China Hospital, Sichuan University (No. 20221216022).

Note that full information on the approval of the study protocol must also be provided in the manuscript.

Plants

Seed stocks	The study did not involve seed.
Novel plant genotypes	The study did not involve plant genotypes.
Authentication	*Describe any authentication procedures for each seed stock used or novel genotype generated. Describe any experiments used to assess the effect of a mutation and, where applicable, how potential secondary effects (e.g. second site T-DNA insertions, mosiacism, off-target gene editing) were examined.*
